# Traffic Risk Environment Impact Analysis and Complexity Assessment of Autonomous Vehicles Based on the Potential Field Method

**DOI:** 10.3390/ijerph191610337

**Published:** 2022-08-19

**Authors:** Ying Cheng, Zhen Liu, Li Gao, Yanan Zhao, Tingting Gao

**Affiliations:** 1 School of Automobile and Transportation, Tianjin University of Technology and Education, Tianjin 300222, China; 2 Hubei Key Laboratory of Power System Design and Test for Electrical Vehicle, Hubei University of Arts and Science, Xiangyang 441053, China; 3 School of Mechanical Engineering, Beijing Institute of Technology, Beijing 100081, China

**Keywords:** autonomous vehicles, traffic safety, environment impact analysis, risk evaluation, potential field theory

## Abstract

Although autonomous vehicles have introduced a promising potential for improving traffic safety and efficiency, ensuring the safety of autonomous vehicles in complex road traffic environments is still a huge challenge to be tackled. To quickly quantify the potential risk factors of autonomous vehicles in traffic environments, this paper focuses mainly on the influence of the depth and breadth of the environment elements on the autonomous driving system, uses the potential field theory to establish a model of the impact of the environmental elements on the autonomous driving system, and combines AHP to quantify equivalent virtual electric quantity of each environment element, so as to realize the quantitative evaluation of the traffic environment complexity. The proposed method comprehensively considers the physical attributes and state parameters of the environmental elements, which compensates for the fact that the shortage of the factors considered in the traffic environment complexity assessment is not comprehensive. Finally, a series of experiments was carried out to verify the reliability of our proposed method. The results show that the complexity of the static elements is determined only by the physical attributes and shape of the obstacle; the complexity of the dynamic elements is determined by the movement of the obstacle and the movement of the autonomous vehicle, and the comprehensive complexity mainly depends on the complexity of their dynamic elements. Compared with other methods, the complexity evaluation values are generally consistent, the absolute percentage error of the majority of samples was within ±5%, and the degree of deviation was −1.143%, which provides theoretical support for autonomous vehicles on safety and the risk assessment in future.

## 1. Introduction

With the continuous development of automobile intelligent technology, autonomous driving is about to enter the comprehensive testing stage, and the demand for testing based on an open road has become increasingly urgent [1,2]. Currently, the most popular testing approach is the Naturalistic-Field Operational Test (N-FOT), which is widely used. However, the limitation of this approach is its low efficiency, because N-FOTs require billions of driving mileages to obtain sufficient “safety evidence” [3,4]. To accelerate the safety testing of autonomous vehicles, scenario-based testing approaches are promising because of the benefits of traffic scenario customization, full coverage of real-world traffic scenarios, and transferable testing results among different regions [5,6]. However, how to ensure the testing scenarios have full coverage for the naturalistic driving conditions and finish the test within limited periods has emerged as a dilemma.

The road traffic environment is an open, nonlinear, random, dynamic system, with high uncertainty, unrepeatable, unpredictable, and inexhaustible characteristic, which is a huge challenge for autonomous driving systems [7,8,9]. The autonomous vehicles should have the ability to assess the complexity level of the current driving scenario and therefore to understand the driving cognitive workload to prevent potential hazard situations. In order to quickly quantify the potential risk factors of autonomous vehicles in traffic environments, complexity is a necessary criticality measure to define the critical scenario, and it is crucial to study how to quantify the complexity of driving scenarios [10,11].

## 2. Related Work

The quantitative assessment of the impact of the traffic environment on the autonomous driving system is still in the exploratory stage. At present, the existing scenario complexity quantification studies mainly have two types of subjective quantification methods and objective quantification methods. Among them, the subjective quantitative methods generally adopt analytic hierarchy process (AHP), information entropy, and so on; for example, Wang et al. [12] established fundamental and additional environment complexity models based on an integrated evaluation method of expert evaluation and the analytic hierarchy process (AHP). Gao X et al. [13] proposed a traffic environment complexity evaluation model based on graphical information entropy, which analyzed the impact of traffic environment on drivers’ mental load, and established a stimulus response model of human driving behavior. This method is highly reliant on human participation and also does not consider the location and speed of dynamic traffic elements.

Meanwhile, the existing objective methods mainly apply data-driven models or machine learning methods on the basis of obtaining the information of the surrounding environment of vehicles; for example, Isabelle Tang et al. [14] proposed a method based on low-cost camera imaging to extract relevant data for the road traffic environmental, and the traffic environment was classified according to the results shown by the data. Zhang H.C. et al. [15] defined the complexity of driving as the cognitive burden of drivers caused by the traffic environment, and the gravity model was applied to evaluate the complexity of the road traffic environment. The concepts of instantaneous complexity, time accumulation complexity, and distance accumulation complexity are proposed in the paper, but the method lacks the calibration of the relevant parameters in the calculation formula and cannot obtain the complexity quantification value directly. Fan N. et al. [16] believed that the driving task complexity originated from instantaneous complexity, and a dynamic complexity measure was proposed based on collision time. At the same time, Yu R.J. et al. [17] proposed to quantify human drivers’ judgement on driving environment complexity, by describing the vehicle–vehicle spatial–temporal interactions from the perspectives of quantity, variety, and relations, but these methods did not consider the complexity of the static traffic environment and had certain limitations to their application [11].

In summary, there are the following problems in the quantitative assessment of traffic environment complexity [18,19,20]. Firstly, the factors considered in the assessment of traffic environment complexity are not comprehensive, the models are relatively simple, and the impact of different environmental factors on driving behavior and complexity is not analyzed from multiple dimensions, such as physical attributes and state parameters [21]. Secondly, previous studies have not made effective use of environmental information extracted from the OpenStreetMap (OSM) domain and prior environmental knowledge, so the real-time online quantitative evaluation of the driving scenarios of autonomous vehicles cannot be conducted [22].

This paper proposes a method to evaluate the complexity of the traffic environment of autonomous vehicles based on the potential field; each environmental element is abstracted as positive point charges or uniformly charged wires, which generates a potential field in regional space, and different electric potential fields can be accumulated and superimposed to form the final environmental potential field, which is used to realize the quantitative assessment of the traffic environment, which can provide theoretical support for the testing of autonomous vehicles.

## 3. Materials and Methods

### 3.1. Definition of Traffic Environment Complexity

The traffic environment is the sum of powers of all the external influences on autonomous driving systems, including road conditions, traffic facilities, land features, meteorological conditions, activities of other traffic participants, and so on. However, these road conditions, facilities, and meteorological conditions vary continuously or discretely within a certain interval, which will lead to endless road traffic scenarios.

**Figure 1:**
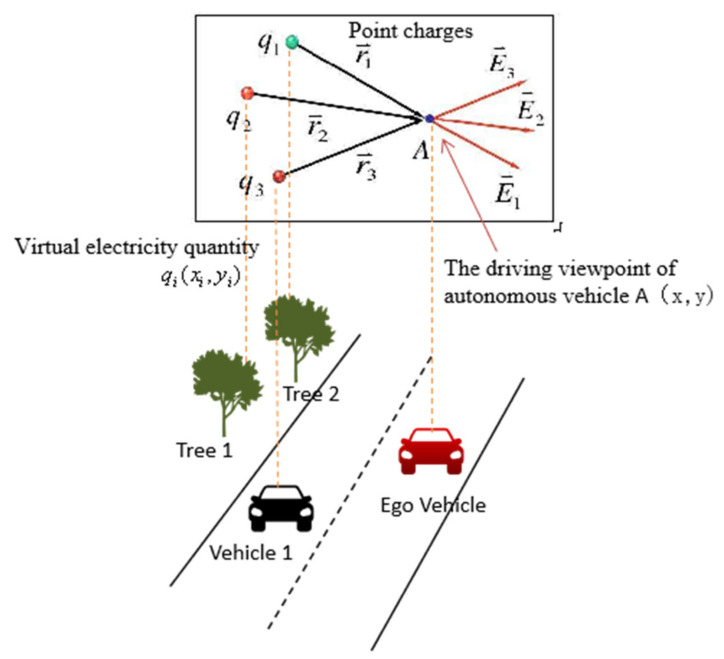
The relationship of the complexity of the traffic environment and the theory of field theory.

Autonomous vehicles can be regarded as an individual in the system. Studying the complexity of the traffic environment is to evaluate the influence of the system on the individual, which is similar to the field theory, as shown in Figure 1.

In this paper, each environmental element is abstracted as positive point charges or uniformly charged wires. According to the field theory [23,24], it generates a potential field in the regional space, which is determined by the category of environmental elements (i.e., virtual electricity quantity) and is inversely proportional to the distance, as shown in formula 1. Meanwhile, different electric potential fields can be accumulated and superimposed to form the final environmental potential field, which is similar to the traffic environmental complexity [22].

(1) $$\begin{array}{l} \vec{E}=\sum_{i}{\vec{E}}_{i}\\ V_{A}=\int_{A\infty }\vec{E}\cdot d\vec{l}\\ =\sum_{i=1}^{n}\int_{A\infty }{\vec{E}}_{i}\cdot d\vec{l}\\ =\sum_{i=1}^{n}V_{i}=\sum_{i=1}^{n}\frac{q_{i}}{4\pi \varepsilon_{0}r_{i}} \end{array}$$

In summary, this paper defines the traffic environment complexity as follows: taking the driving viewpoint of the autonomous vehicle (i.e., the elliptical viewpoint of the human driver’s eye) as the coordinate reference, calculating the static element complexity and dynamic element complexity based on the viewpoint scene, and finally weighting the static element complexity and dynamic element complexity to obtain the traffic environment complexity. Therefore, the complexity assessment of traffic environment is an objective description of the impact of the environmental elements on the autonomous driving system based on the viewpoint scene. The road traffic environment complexity is an objective property, which is determined by the physical characteristics of the environmental elements and does not vary with the capacity of the testing vehicle [25,26,27,28].

### 3.2. The Calculation Model of the Traffic Environment Complexity

#### 3.2.1. Static Elements Complexity

Firstly, considering the static environmental elements, such as traffic signs, plants, markings, ancillary facilities, etc., each environmental element is abstracted as a positive point charge, and then we use the artificial potential field to calculate the static element complexity. Taking the static element $q_{J}^{i}(x_{i},y_{i})$ as an example, it is known that the potential field based on the viewpoint coordinates A(x,y) of the tested autonomous vehicle is expressed as follows:

(2) $$C_{J}=\sum_{i=1}^{n}\frac{q_{J}^{i}}{4\pi \varepsilon_{0}r_{J}^{i}}$$

(3) $$r_{J}^{i}=\sqrt{{\left(x_{i}-x\right)}^{2}+{\left(y_{i}-y\right)}^{2}}$$

where $q_{J}^{i}$ is the virtual electricity quantity of the *i*th static element, which is determined by the static element category, and $r_{J}^{i}$ is the distance from the viewpoint coordinate to the center of the *i*th static element and *n* is the number of static environment elements.

Considering that the actual obstacles all have a certain volume, and when the distance is small to a certain extent in physics, the formula no longer holds, we introduce *r*_0_ to denote the equivalent radius of the obstacle, and the distance calculation formula is modified as follows:

(4) $$r_{J}^{i}=\max(\sqrt{{\left(x_{i}-x\right)}^{2}+{\left(y_{i}-y\right)}^{2}},r_{0})$$

Furthermore, considering the linear environmental elements, e.g., lane boundary, traffic guardrail, and lane line, etc., each environmental element is abstracted as an uniformly charged wire, and we use the artificial potential field to calculate the static element complexity. Taking the lane line $L_{I}$ as an example, assuming that the equation of its central reference line is ax+by+c=0, then $\left|r_{J}^{i}\right|$ is the distance from the point to the lane line, so a positive real number *r*_0_ is introduced to modify the distance calculation formula.

(5) $$\left|r_{J}^{i}\right|=\max(\frac{\left|ax+by+c\right|}{\sqrt{a^{2}+b^{2}}},r_{0})$$

Similarly, when the type of the lane line is a curve, taking the circular curve ${\left(x-x_{i}\right)}^{2}+{\left(y-y_{i}\right)}^{2}=R^{2}$ as an example, the calculation formula of the environmental potential field is the same as above, and the distance calculation formula is:

(6) $$\left|r_{J}^{i}\right|=\max(\left|\sqrt{{\left(x_{i}-x\right)}^{2}+{\left(y_{i}-y\right)}^{2}}-R\right|,r_{0})$$

#### 3.2.2. Dynamic Elements Complexity

Considering dynamic environmental elements, such as pedestrians, vehicles, non-motorized vehicles, etc., we then use the artificial potential field to calculate the dynamic element complexity. Taking the static element $q_{D}^{i}(x_{i},y_{i})$ as an example, it is known that the potential field based on the viewpoint coordinates A(x,y) of the tested autonomous vehicle is expressed as follows:

(7) $$C_{D}=\sum_{i=1}^{n}\frac{\omega_{D}^{i}\cdot q_{D}^{i}}{4\pi \varepsilon_{0}r_{D}^{i}}$$

(8) $$r_{D}^{i}=\max(\sqrt{{\left(x_{i}-x\right)}^{2}+{\left(y_{i}-y\right)}^{2}},r_{0})$$

where $q_{D}^{i}$ is the virtual electric quantity of the *i*th dynamic element, which is determined by the dynamic element category, $r_{D}^{i}$ is the distance from the viewpoint coordinate to the center of the *i*th dynamic element, $\varpi_{D}^{i}$ is the proportional enhancement factor, and *n* is the number of dynamic environment elements.

Regarding the proportional enhancement factor $\varpi_{D}^{i}$, according to human driving habits and subjective feelings, when the environmental elements are in motion, the influence of the objects located in the area in front of them will be strengthened and the influence of the objects located in the area behind them should be weakened, which is similar to the Doppler effect. Therefore, the proportional enhancement factor $\varpi_{D}^{i}$ is calculated as follows:

(9) $$\varpi_{D}^{i}(x,y)=\frac{c}{\sqrt{{\left(c\cdot \cos\theta -v_{ix}\right)}^{2}+{\left(c\cdot \sin\theta -v_{iy}\right)}^{2}}}$$

(10) $$\sin\theta =\frac{y-y_{i}}{\sqrt{{\left(x_{i}-x\right)}^{2}+{\left(y_{i}-y\right)}^{2}}}$$

(11) $$\cos\theta =\frac{x-x_{i}}{\sqrt{{\left(x_{i}-x\right)}^{2}+{\left(y_{i}-y\right)}^{2}}}$$

where *c* is the propagation velocity of the potential field, which is a constant to be calibrated (the value is not less than the maximum speed of vehicle), θ is the directional angle of the $r_{D}^{i}$, and $v_{ix}$ and $v_{iy}$ are the decomposition quantities of the velocity in the *X* and *Y* axis for the dynamic elements.

Other than when calculating the environment complexity, we should consider not only the vehicles and pedestrians in this lane, but also the vehicles and pedestrians in other lanes. The difference between the two is that the effect of the latter on the autonomous vehicles is smaller than the effect of the former on the autonomous vehicles; the more lanes are separated, the smaller the effect is.

In order to quantify the environmental elements complexity located in different lanes, an “electron energy level” model is introduced; each lane is considered as an energy level track, and different lanes have different energy levels. The lane in which the Ego vehicle is located is defined as $L_{1}$ and its energy level is $\;E_{1}$, and the energy level $E_{n}$ of the lane $L_{n}$, which is a lane of *n* lanes separated from lane $L_{1}$*,* is expressed as follows:

(12) $$E_{n}=\frac{E_{1}}{n^{2}}$$

Therefore, the environmental potential field generated by the dynamic environmental elements of lane $\;L_{n}$ is expressed as follows:

(13) $$C_{D}=\sum_{i=1}^{n}\frac{\varpi_{D}^{i}\cdot q_{D}^{i}}{4\pi \varepsilon_{0}r_{D}^{i}n^{2}}$$

#### 3.2.3. Complexity of the Traffic Environment

Given that different potential fields can accumulate and form the final environmental potential field, the complexity of static elements and dynamic elements are weighted and summed to obtain the traffic environmental complexity $C_{E}$. The traffic environmental complexity is given as follows:

(14) $$C_{E}=\alpha \cdot C_{J}+\beta \cdot C_{D}$$

where $C_{E}$ is the traffic environment complexity based on the viewpoint coordinates (*x, y*), α, β is the weights of static element complexity and dynamic element complexity; here, the value of α*,* β is 0.35 and 0.65.

Based on the values of $C_{E}$, the traffic environment complexity for autonomous vehicles is graded as follows:

When 80 ≤$\;C_{E}\;$≤ 100, the level of the traffic environment complexity for autonomous vehicles is extremely complex.

When 60 ≤$\;C_{E}\;$< 80, the level of the traffic environment complexity for autonomous vehicles is more complex.

When 40 ≤$\;C_{E}\;$< 60, the level of the traffic environment complexity for autonomous vehicles is average.

When 0 <$\;C_{E\;}$< 40, the level of the traffic environment complexity for autonomous vehicles is simple.

### 3.3. Determination of Equivalent Virtual Electric Quantity of Traffic Environment Elements

The above virtual electricity quantity is mainly determined by the category of environmental elements, which represent the impact of the element on self-driving vehicles. Strictly speaking, the virtual electricity quantity of each environment element is different, but the virtual electric quantity of similar environmental elements should be approximately equal. So, in order to have convenient calculations, we divide the environmental elements into several categories, mainly including several categories of humans, motor vehicles, animals, green plants, ancillary facilities, signs, and marking lines.

The virtual electric quantity of the above elements categories was calibrated using analytic hierarchy process (AHP), and the judgment matrix was constructed by comparing the relative importance of each other using the 9-scale method as seen in Table 1.

Secondly, the maximum eigenvalue and the corresponding eigenvector were calculated.

$$\lambda_{max}=7.1279\;w=\left[0.7475,0.5088,0.3407,0.1306,0.968,0.1900,0.608l\right]$$

Finally, a consistency test was performed.

(15) $$CI=\frac{\lambda -n}{n-1}=\frac{7.1279-7}{7-1}=0.0213$$

Checking the table and the random consistency index *RI* was 1.32, the consistency ratio was calculated as follows:

(16) $$CR=\frac{CI}{RI}=0.0802<0.1$$

After the consistency test, here the feature vector *w* refers to the virtual electric quantity of these several categories of elements, as shown in Table 2.

## 4. Validation and Discussion

### 4.1. Verification of Complexity Calculation in Virtual Traffic Environment

Several traffic environment simulation scenarios were built based on the PreScan software, and the verification of the traffic environment complexity model in different scenarios was carried out.

#### 4.1.1. Complexity Calculation of Off-Road Traffic Environment

**Figure 2:**
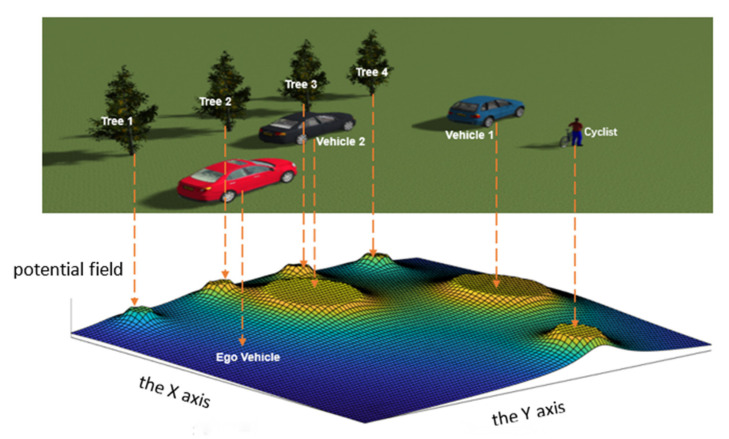
The results of potential field of the off-road road traffic environment.

The off-road road traffic environment refers to the absence of roads and lane lines within the traffic area of the region, as shown in Figure 2. Taking the autonomous vehicle as the Ego Vehicle perspective, the traffic environment complexity is calculated and the results of environmental potential field are visualized as follows.

The traffic environment complexity $C_{E}$ is generated by one cyclist, two vehicles, and four trees; it can be seen that each “peak” is produced by the corresponding environmental element and “peak height” is determined by the category of environmental elements. Generally speaking, the “peak height” generated by vehicles and pedestrians is higher than other environmental elements, and the “peak radius” is determined by the equivalent size of environmental elements, and obviously the “peak radius” of vehicles is larger than that of pedestrians and trees.

#### 4.1.2. Complexity Calculation of Highway Traffic Environment

The traffic environment of highway means that the meaningful traffic elements such as road boundaries and lane lines not only have a restraining effect on moving vehicles, but also on other traffic participants. Therefore, this paper defines the constraint of the road boundary as a strong constraint. Here, by setting its the virtual electric quantity, the potential field at the road boundary is set as the maximum and the potential field generated by all the traffic elements outside the road can be ignored; that is, the road boundary has a “shielding effect” on the environmental elements outside the road. Accordingly, the lane line restraint is set as a weak restraint, i.e., drivers should avoid driving on the lane line and changing lanes as often as possible, but it is not absolutely prohibited.

**Figure 3:**
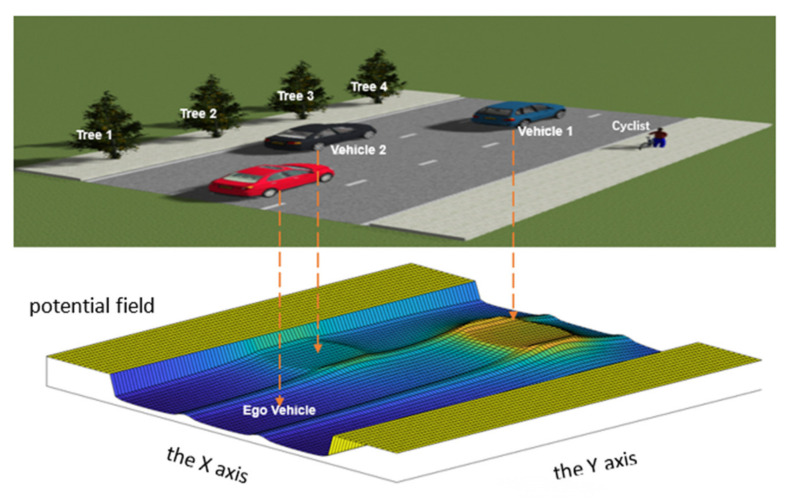
The results of potential field of the highway traffic environment.

When roads and lane lines exist in the environment, the potential field of the environmental elements is shown in Figure 3. In the figure, the blue vehicle 1 is in the same lane as the Ego Vehicle, and the black vehicle 2 is in the adjacent lane of the Ego Vehicle. The potential energy field generated by the black vehicle 2 is one-quarter of the potential energy field generated by the blue vehicle 1, and the “peak” generated by the black vehicle in the figure becomes lower. Both cyclists and trees are disregarded because they are outside the lane line.

#### 4.1.3. Complexity Calculation of Urban Road Environment

The traffic environment of the urban road refers to a more complex traffic scene that contains dynamic traffic elements, such as pedestrians and vehicles, and usually also static traffic elements such as traffic lights, stop lines, and crosswalks.

In the actual traffic environment, intersections as a relatively open area, the behavior of the traffic participants has great uncertainty and complexity, so that all the other traffic participants in the region need to be taken into account when calculating the complexity of the traffic environment. However, under the strong constraint of traffic signal lights, when there is no cross conflict point with some vehicles, the energy generated by other vehicles should be attenuated.

**Figure 4:**
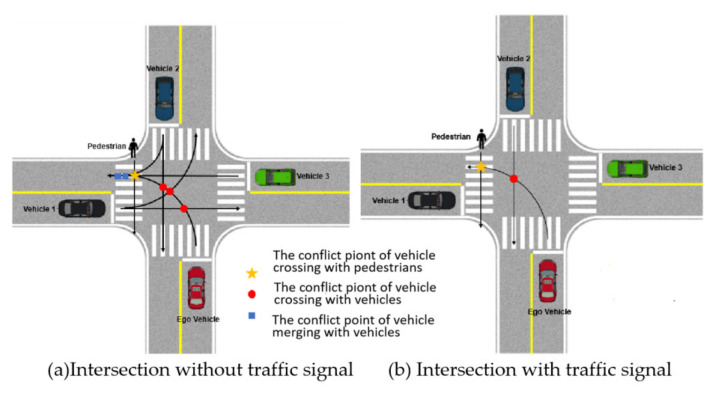
The conflict points formed in the left turn process of self-driving vehicles at intersections.

Taking the intersection without traffic signals (Figure 4a) and with traffic signals (Figure 4b) as examples, the conflict points formed in the left turn process of self-driving vehicles are shown in Figure 4.

**Figure 5:**
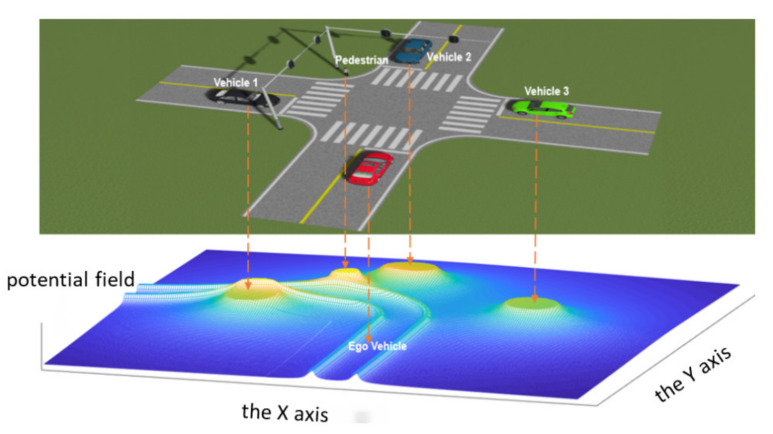
The potential field of the environmental elements at intersection without traffic signal.

At the intersection without traffic signal, the potential fields generated by vehicle 1, vehicle 2, vehicle 3 and pedestrians all should be taken into account when calculating the environment complexity of the autonomous vehicle, the potential field of the environmental elements is shown in Figure 5.

**Figure 6:**
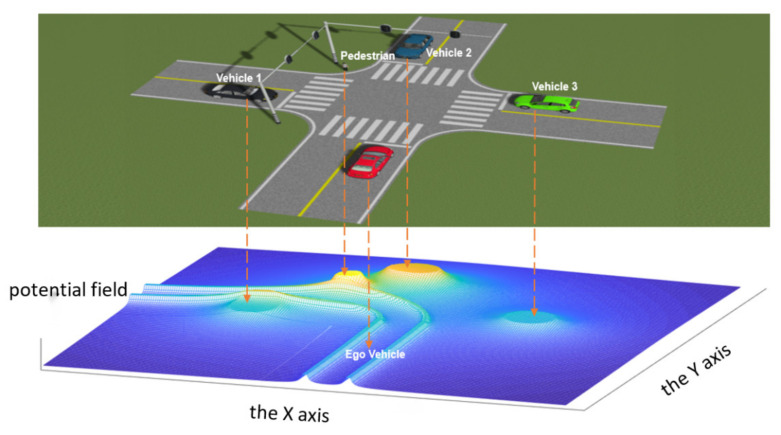
The potential field of the environmental elements at intersection with traffic signal.

Compared with the intersection without traffic signal, the intersections with traffic signal have the strong constraint effect of traffic signal lights, Vehicles 1 and 3 have no traffic conflicts with the autonomous vehicle; thus, it is necessary to attenuate the potential field generated by them. The potential field of each environmental element at the intersection is shown in Figure 6. The “peak” of the potential energy field generated by Vehicles 1 and 3 becomes lower than that of Vehicle 2 and the pedestrian.

### 4.2. Verification of Complexity Calculation in Real Traffic Environment

This paper selects the Huawei autonomous driving video in a real traffic environment for the complexity calculation of traffic environment elements, and the part of environmental element information parameters collected by the system and the element ID of the complexity calculation are shown in Table 3.

**Figure 7:**
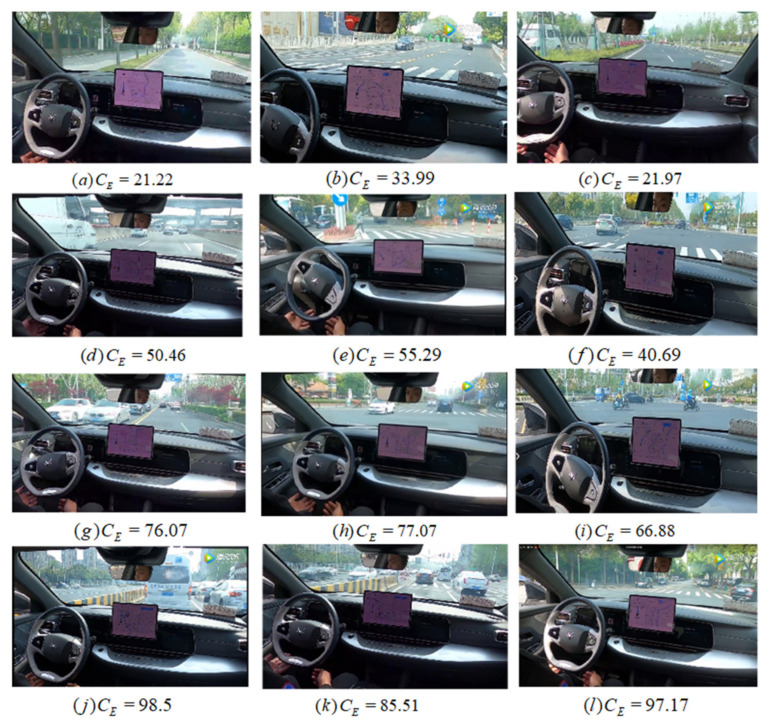
The experimental results of four different complexities.

Figure 7 shows the results of experiments in four different complexities. Road data are divided into four levels based on the complexity value: simple data whose complexity is between 0 and 40, general data for which the complexity is between 40 and 60, medium data for which the complexity is between 60 and 80, and extreme data for which the complexity is between 80 and 100. It can be seen that the complexity of the traffic environment shown in (a)–(c) is simple, (d)–(f) is generally complex, (g)–(i) is more complex, and (j)–(l) is extremely complex. We can find that the calculated complexity is consistent with the complexity of the scene.

**Figure 8:**
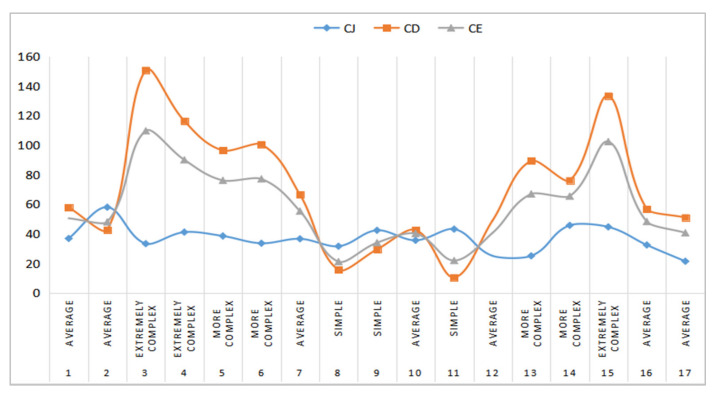
The complexity statistical analysis of static element complexity, dynamic element complexity, and comprehensive complexity.

We randomly selected 17 traffic scenarios for the complexity statistical analysis; their static element complexity $\;C_{J}$, dynamic element complexity $\;C_{D}$, and comprehensive complexity $C_{E}$ are shown in Figure 8. As can be seen from the figure, the static element complexity of different traffic scenes is relatively small and close, and their value stays around 20 to 60. However, the dynamic element complexity of different traffic scenes varies greatly, and their values vary from 20 to 150. It can be seen that the comprehensive complexity mainly depends on depends on the dynamic element complexity. Obviously, those scenarios with high complexity are mainly due to the great dynamic element complexity, as shown in Figure 7j–l, and those scenarios with low complexity are mainly due to the relatively small dynamic element complexity, as shown in Figure 7a–c.

### 4.3. Comparative Analysis with the Other Methods

**Figure 9:**
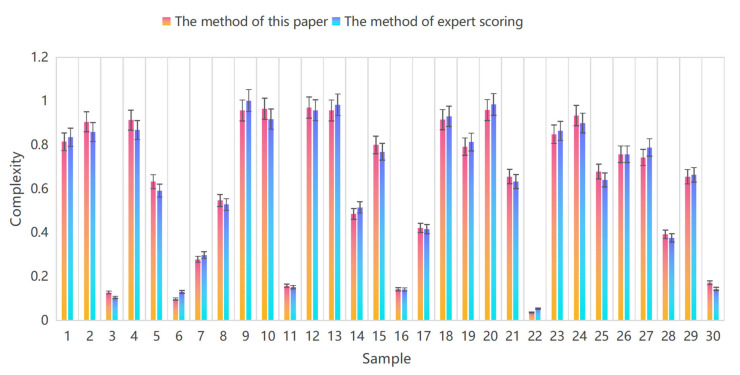
The analysis comparing the methods of this paper and the evaluation method of expert scoring.

To further verify the efficiency of the proposed model, it is compared with the evaluation method of expert scoring based on complexity. Thirty groups of real traffic scene videos and photos were selected for the experiments, two complexity evaluation methods were used to calculate their values, and the results are shown in Figure 9. The results show that the complexity evaluation values obtained by the two methods are generally consistent, the absolute percentage error of the majority samples is within ±5%, and the degree of deviation is −1.143%. This shows that the established prediction model error is within the expected range and can more accurately characterize the relationship between environmental elements and the complexity of the traffic environment.

## 5. Conclusions and Future Work

Scenario-based testing and evaluation technology has become the key to autonomous vehicles driving safely on the road. An advanced autonomous vehicle should be able to evaluate the complexity of the current driving scenario and drive the vehicle properly to prevent potential hazardous situations. However, a complex testing scenario can benefit the validation and verification of autonomous vehicles. The quantification of scenario complexity can both enhance the environmental cognition and accelerate the testing and validation work of autonomous vehicles [29,30].

(1) This paper defines the traffic environment complexity as follows: taking the driving viewpoint of the autonomous vehicle (i.e., the elliptical viewpoint of the human driver’s eye) as the coordinate reference, calculating the static element complexity and dynamic element complexity based on the viewpoint scene, and finally weighting the static element complexity and dynamic element complexity to obtain the traffic environment complexity.
(2) The traffic environment is the sum of powers of all the external influences on autonomous driving systems; this paper mainly focuses on the influence of the depth and breadth of the environment elements on the autonomous driving system, uses the potential field theory to establish the impact model of the environment elements on the autonomous driving system, and combines AHP to quantify equivalent virtual electric quantity of each environment element, so as to realize the quantitative evaluation of the traffic environment complexity.
(3) A series of experiments was carried out to verify the reliability of our proposed method. The results show the static elements complexity is determined only by the physical attributes and shape of the obstacle, the dynamic elements complexity is determined by the movement of the obstacle and the movement of the autonomous vehicle, and the comprehensive complexity depends mainly on their dynamic elements complexity. Compared with other methods, the complexity evaluation values are generally consistent, the absolute percentage error of the majority of samples are within ±5%, and the degree of deviation was −1.143%; this provides theoretical support for autonomous vehicles on-line testing and risk assessment in future. Scenario complexity quantification is the way to hierarchize the standard scenarios. It helps researchers to build hierarchical benchmarks and develop autonomous vehicles at different levels.

## Figures and Tables

**Table 1:** Judgment matrix.

Elements	A1	A2	A3	A4	A5	A6	A7
Humans A1	1	2	3	5	6	4	9
Motor Vehicles A2	1/2	1	2	4	5	3	8
Animals A3	1/3	1/2	1	3	4	2	7
Green Plants A4	1/5	1/4	1/3	1	2	1/2	2
Ancillary Facilities A5	1/6	1/5	1/4	1/2	1	1/2	2
Signs A6	1/4	1/3	1/2	2	2	1	3
Marking Lines A7	1/9	1/8	1/7	1/2	1/2	1/3	1

**Table 2:** The virtual electricity quantity of each category of elements.

Element Classification	Humans	Motor Vehicles	Animals	Green Plants	Ancillary Facilities	Signs	Line Marking
Name	Pedestrian Cyclist Non-motorized vehicles	Car Bus Truck	Poultry Livestock Wildlife	Trees Shrubs Semi-shrubs	Telegraph Pole Isolation Strip Traffic Guardrail	Warning Signs Prohibition Signs Indicator Signs	White Lane Lines Yellow Lane Lines —
virtual electricity quantity	0.7475	0.5088	0.3407	0.1306	0.0968	0.1900	0.0608

**Table 3:** The environmental element information parameters and the element ID of the complexity calculation.

Order	Environment Element ID for Complexity Calculations	Category of Environmental Elements	Name of Environment Elements	Virtual Power Quantity	Distance/m	Angle/°
1	A40206	General road guide sign	Cross intersection informed sign	18	38	0
2	A30102	Indication sign	Sign of Turning left	5	13	0
3	B20102	Prohibit marking line	Double-yellow solid line	16	0.7	−90
4	B10106	Indicate marking line	White dotted line at the roadway edge	12	0.3	90
5	C50304	Green plants	Bush	8	4.5	90
6	D30300	Vehicle	Vehicle1	22	35	0
7	D30300	Vehicle	Vehicle2	22	32	12
8	D30300	Vehicle	Vehicle3	22	4	−29
9	D30300	Vehicle	Vehicle4	22	4.5	−41
10	D30300	Vehicle	Vehicle5	22	8	−20
11	D30300	Vehicle	Vehicle6	22	12	−16

## Data Availability

The datasets used and analyzed during the current study are available from the corresponding author on reasonable request.
